# Migraine Frequency Decrease Following Prolonged Medical Cannabis Treatment: A Cross-Sectional Study

**DOI:** 10.3390/brainsci10060360

**Published:** 2020-06-09

**Authors:** Joshua Aviram, Yelena Vysotski, Paula Berman, Gil M. Lewitus, Elon Eisenberg, David Meiri

**Affiliations:** 1 Faculty of Biology, Technion-Israel Institute of Technology, Haifa 3200003, Israel; shukiaviram@gmail.com (J.A.); lalatrololo@gmail.com (Y.V.); bermansh@gmail.com (P.B.); lgil@technion.ac.il (G.M.L.); 2 Institute of Pain Medicine, Rambam Health Care Campus, Haifa 3109601, Israel; e_eisenberg@rambam.health.gov.il; 3 Rappaport Faculty of Medicine-Technion, Israel Institute of Technology, Haifa 3525433, Israel

**Keywords:** cannabinoids, migraine: chronic pain, opioids, triptans, disability

## Abstract

Background: Medical cannabis (MC) treatment for migraine is practically emerging, although sufficient clinical data are not available for this indication. This cross-sectional questionnaire-based study aimed to investigate the associations between phytocannabinoid treatment and migraine frequency. Methods: Participants were migraine patients licensed for MC treatment. Data included self-reported questionnaires and MC treatment features. Patients were retrospectively classified as responders vs. non-responders (≥50% vs. <50% decrease in monthly migraine attacks frequency following MC treatment initiation, respectively). Comparative statistics evaluated differences between these two subgroups. Results: A total of 145 patients (97 females, 67%) with a median MC treatment duration of three years were analyzed. Compared to non-responders, responders (*n* = 89, 61%) reported lower current migraine disability and lower negative impact, and lower rates of opioid and triptan consumption. Subgroup analysis demonstrated that responders consumed higher doses of the phytocannabinoid ms_373_15c and lower doses of the phytocannabinoid ms_331_18d (3.40 95% CI (1.10 to 12.00); *p* < 0.01 and 0.22 95% CI (0.05–0.72); *p* < 0.05, respectively). Conclusions: These findings indicate that MC results in long-term reduction of migraine frequency in >60% of treated patients and is associated with less disability and lower antimigraine medication intake. They also point to the MC composition, which may be potentially efficacious in migraine patients.

## 1. Background

Chronic migraine constitutes a disabling neurological disorder, affecting around one to two percent of the global population worldwide [1]. Traditionally, abortive migraine treatments include triptans [2], non-steroidal anti-inflammatory drugs (NSAIDs) [3], paracetamol [4], ergots [5], opioids [6], and antiemetics [7]. Preventive treatments include antidepressants, anticonvulsants, beta-blockers, and more recently, anti-calcitonin gene-related peptide (CGRP) agents [8]. In recent years, the use of medical cannabis (MC) for the treatment of chronic pain in general has emerged, along with an increase in demand and use by migraine patients. A recent cross-sectional study found that nearly 36% of MC users reported using it to treat headache and migraine [9]. An additional survey reported about 50% reduction of migraine and headache severity following inhaled cannabis consumption [10]. Nevertheless, good clinical data supporting the beneficial effect of MC on migraine are scarce.

Both clinical and preclinical data suggest an abnormal endocannabinoid system function in migraine. In patients with chronic migraine, the cerebral spinal fluid (CSF) concentrations of anandamide (AEA) were significantly lower and the concentrations of palmitoylethanolamide (PEA) were significantly higher compared to non-migraine headache patients and controls [11]. Furthermore, reduced levels of AEA degrading enzymes were found in platelets of patients with chronic migraine [12]. In animal models of migraine, administration of AEA diminished hyperalgesic behavior [13], and the plant-derived (-)-Δ^9^-*trans*-tetrahydrocannabinol (THC) showed anti-migraine effects in rats [14]. Whilst the available evidence suggests involvement of the endocannabinoid system and a potential for MC treatment to be therapeutic in migraine, more research is required to demonstrate the efficacy parameters of MC treatment for migraine. The complexity of the MC plant and how to design therapeutics from it must also be considered.

The single-compound, single-target approach in pharmaceutical science is a long-standing tradition embedded in our approach to clinical problem-solving. This is wholly different to MC treatment, which is often times multi-compound, whole-plant treatment. The cannabis plant contains hundreds of different active components, including phytocannabinoids, terpenes, and flavonoids [15]. While THC and cannabidiol (CBD) are among the most well-known phytocannabinoids, others are likely to have biological activity as well [16]. Hence, it is conceivable that various combinations of phytocannabinoids differ in their anti-migraine activity. This multi-compound effect of cannabis has been called the “entourage effect” [17], which suggests that studies examining the role of single-molecule cannabinoids in disease may not necessarily capture the synergy at play in multi-compound MC treatment. To add to the complexity of MC treatment with multiple compounds, there are hundreds of different cannabis cultivars, each with its own unique chemical composition [18]. Recently, we have developed an electrospray ionization mass spectrometry - liquid chromatography mass spectrometry (ESI-LC/MS) approach for comprehensive identification and quantification of phytocannabinoids in cannabis. We have identified over 90 phytocannabinoids, of which approximately 20 were previously unknown [19]. Quantifying the multitude of phytocannabinoids is the first step to better understanding the therapeutic potential of each cannabis cultivar, and therefore how to plan better clinical studies.

The regulations that govern cannabis use for medical purposes in Israel under the Israeli Ministry of Health (IMOH) allow only specific indications for which a patient can be issued with a MC license by their prescribing physician [20]. Whilst migraine is not an approved indication, it is sometimes comorbid with approved indications, such as gastrointestinal disease and chronic neuropathic non-cancer pain. In the case of chronic non-cancer pain, migraine is itself sometimes characterized as a chronic non-cancer pain condition, depending on its frequency and duration. In order to receive a license, the Medical Cannabis Unit (MCU) of the IMOH reviews MC license applications and provides the physician with either an approval or refusal, along with the justification for all declined applications.

Applications to the MCU include recommendations on MC routes of administration (oil extracts for sublingual use or inflorescence for inhalation and vaporization) and the starting monthly dose of 20 g (MCU approval is required for any increased dose). The physician will then recommend a specific MC cultivar or combinations of cultivars to their patients; however, the patient ultimately makes the final decision of which cultivar(s) to consume. In order for patients to determine which cultivar(s) best meets their therapeutic needs, they conduct a personal trial-and-error process. In addition, the guidelines for titration schedules, which are delivered as recommendations either by a nurse or by instructors from the companies licensed to cultivate cannabis, are not enforced. Titration scheduling covers doses per day, recommended starting dose, guidelines for increasing or decreasing the dose, and the maximum allowable dose. This means that the doses of phytocannabinoids consumed by the patient are not controlled.

The purpose of this cross-sectional study was to calculate the total dose of individual phytocannabinoids consumed by migraine patients and explore differences in dosages between subgroups of patients according to their changes in frequency of migraine attacks. Additionally, associations between changes in frequency of migraine attacks to migraine disability severity, sleep quality and timing, and migraine analgesics consumption were explored.

## 2. Materials and Methods

### 2.1. Subject

Patients were eligible to participate in this study if they were Hebrew speaking, aged ≥18 years with a standing MC license for the treatment of any approved condition, coupled with a diagnosis of comorbid migraine by their physician.

### 2.2. Study Procedure

Data were collected after the study was approved by the Institutional Ethics Committee of the Technion, Institute of Technology, Haifa, Israel (#011-2016). An existing database of Israeli patients with a MC license (*n* = 3218) was used to contact those patients who fulfilled the eligibility criteria for this cross-sectional study. Patients who had elected to disclose their email address for future studies and who also reported a diagnosis of migraine (*n* = 423, 13%) were invited to complete an online questionnaire after reading an explanation of the study. Prior to completing the questionnaire, the patients became participants after confirming their migraine diagnosis was received by a physician and after they signed an electronic consent form. Data were collected between August 2019 and February 2020. Participants were not offered financial compensation. While questionnaire data were being collected, the most prominent and most frequently administered cultivars from various approved cultivators in Israel were analyzed for phytocannabinoid content by ESI-LC/MS. Importantly, the chemical analyzes were performed on inflorescence cultivars, which were received from the cultivators only and not directly from the patients. Due to normal variation in chemical constituents of plant material and the expected variability between the cultivars analyzed in the lab compared to those consumed by patients, only phytocannabinoids that were consumed with minimum average concentrations of 0.1 g per month were analyzed. The individual phytocannabinoid monthly dose was calculated for each patient.

### 2.3. Study Questionnaires

Online questionnaire data were collected by secure survey technology Qualtrics^®^ (Provo, Utah, version 12018) [21]. Questionnaires consisted of demographic information, including age, gender, MC treatment duration (years), and BMI. Data on migraine characteristics included the number of migraine days in the last month and the month prior to MC treatment initiation; age of migraine initiation; average current duration of a migraine attack (hours); and the presence of aura, nausea or vomiting, photo- or phonophobia, uni- or bilateral manifestation, and aggravation during physical activity of the migraine attack. Information on the analgesics and the specific abortive or preventive migraine medications was collected. Validated questionnaires included the migraine index disability scale (MIDAS) [22], the headache impact test (HIT-6) [23], and the Pittsburgh sleep quality index (PSQI) [24]. Additionally, MC treatment characteristics included administration route, cultivar name, cultivator brand, total monthly dose (grams), monthly dose of each specific cultivar (grams), and related adverse effects (AEs).

### 2.4. Phytocannabinoid Profiling of Cannabis Cultivars

Air-dried medical cannabis cultivars were obtained from several Israeli medical cannabis cultivators. Reagents, analytical standards, and general methodologies for phytocannabinoid extraction and analysis from cannabis were conducted according to our previously published methods [18,19].

Briefly, for phytocannabinoid extraction, 100 mg of ground cannabis inflorescences were accurately weighed and extracted with 1 mL ethanol. Samples were agitated in an orbital shaker at 25 °C for 15 min and then centrifuged at 4200 rpm. A fraction of the supernatant was collected and filtered through a 0.22 µm PTFE syringe filter and diluted at ratios of 1:9, 1:99, and 1:999 *v*/*v* cannabis extract to ethanol. Phytocannabinoid analyses were performed using a Thermo Scientific ultra-high-performance liquid chromatography (UHPLC) system coupled with a Q Exactive™ Focus Hybrid Quadrupole Orbitrap mass spectrometer (MS, Thermo Scientific, Bremen, Germany). The chromatographic conditions were as detailed in Baram et al. (2019) [18]. Identification and absolute quantification of phytocannabinoids was performed by external calibrations [19]. Compounds for which there were no analytical standards commercially available were semi-quantified [19]. For each phytocannabinoid, the concentrations of the acid and its neutral counterpart were summed and reported as the total content. For example, the concentration of total THC was calculated as ${\mathrm{Total}\;}^{}\mathrm{THC}=\mathrm{THCA}\;\times \;0.877+\mathrm{THC}$. Here, 0.877 is the molar ratio between the two compounds, which corrects for a change in the mass of (-)-Δ^9^-*trans*-tetrahydrocannabinolic acid (THCA) as a result of decarboxylation. For compounds with no absolute identification, neutral or acid concentrations were utilized.

### 2.5. Statistical Analysis

R software (V.1.1.463) with tidyverse [25], pheatmap [26], and atable [27] packages were used to analyze differences in outcome measures by Pearson’s chi-square test for categorical measures and Kruskal–Wallis rank sum test for numeric measures. For the effect size (i.e., odds ratio, OR) and confidence interval (CI), we utilized Cohen’s d test. As is customary in recent migraine clinical trials [28], the primary outcome of this study was the clinically significant reduction in the monthly frequency of migraine attacks following the initiation of MC treatment (i.e., ≥50%; responders) compared to non-responders (i.e., <50%). Shapiro–Wilk test of normality demonstrated non-normal distribution for all measures; thus, data are presented as the median and lower and upper quartiles (Q1–Q3). Differences were considered significant at the *p* < 0.05 level. Incidences are presented as the number and percentage of patients.

## 3. Results

### 3.1. Subjects

We established a patient-reported outcomes database of Israeli patients with a preexisting MC license for various MCU-approved indications (*n* = 3218); the specific data in this database were previously described [29]. A total of 423 (13%) patients reported receiving a diagnosis of migraine in this database population. These patients’ reasons for MC license approval was chronic neuropathic non-cancer pain (81%), cancer-related disorders (9%), post-traumatic stress disorders (7%), gastrointestinal disorders (2%), and neurological disorders (1%). A total of 231 (54% response rate) patients responded to participate in the current study.

A total of 145 patients reported on both the monthly frequency of migraine attacks before and after MC treatment initiation; these patients represent the sample that is analyzed and reported in this paper. The sample consisted of a majority of females (*n* = 97, 67%), with a median age of 45 (34–54). These patients were treated with MC for over a year (3 (2.4–4.6) years), with a range of MC treatment from one to 12 years (Table 1). Notably, no significant differences were found between responders and non-responders in the demographic and MC treatment measures.

### 3.2. Migraine and Sleep Features

We divided our sample into non-responders (i.e., <50%; *n* = 56, 39%) and responders (i.e., ≥50%; responders *n* = 89, 61%) based on their reduction of monthly frequency of migraine attacks from pre-MC to the current post-MC period. No significant difference was found in monthly migraine attack frequency prior to MC treatment initiation (15 (7.8–30) and 14 (8–27), respectively) (0.06 95% CI (−0.27 to 0.41); *p* = 0.71), strengthening the division methodology, as both subgroups started from a similar standpoint. Moreover, there were no significant differences between the subgroups in any of the current migraine features, including the age of migraine diagnosis, average duration of migraine attacks, activity-induced aggravation of migraine, unilateral migraine, bilateral migraine, presence of aura prior to migraine, nausea during migraine, or phono- or photophobia during migraine (Table 2).

**Figure 1:**
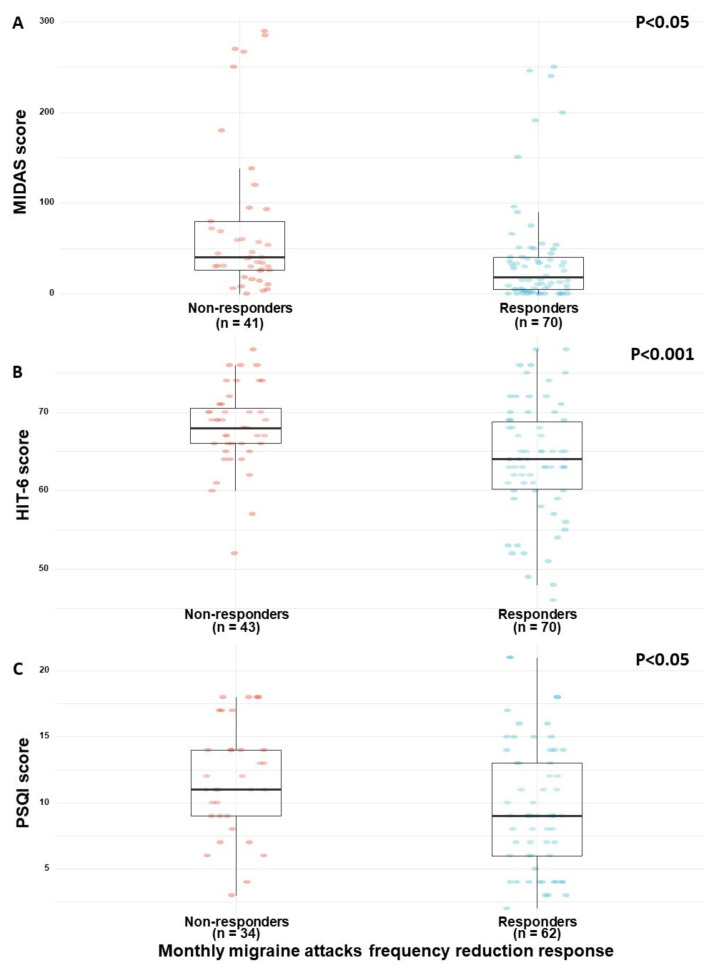
(**A**–**C**) Clinical differences between responders and non-responders. Note: MIDAS, migraine index disability scale; HIT-6, headache impact test; PSQI, Pittsburgh sleep quality index. Response refers to reduction in the monthly frequency of migraine attacks following the initiation of MC treatment (i.e., ≥50%) compared to non-responders (i.e., <50%).

We found that responders were more likely to report lower MIDAS (Figure 1A) and HIT-6 (Figure 1B) questionnaires scores (18 (5–40) and 64 (60–69), respectively) than non-responders (40 (26–80) and 68 (66–70), respectively) (0.50 95% CI (0.11 to 0.90); *p* < 0.05 and 0.66 95% CI (0.26 to 1.00); *p* < 0.001, respectively). Moreover, responders reported better sleep quality (9 (6–13)) than non-responders (11 (9–14)) (0.46 95% CI (0.03 to 0.89); *p* < 0.05)) (Figure 1C). Nonetheless, the evaluated sleep timing measures of sleep latency and sleep duration did not vary significantly between the migraine response subgroups (Table 3).

### 3.3. MC Treatment Safety

MC-related adverse effects (AEs) were reported by 37% (*n* = 53) of the sample. Notably, non-responders reported higher incidences of any AEs (*n* = 26, 46%) than responders (*n* = 27, 30%) (0.46 95% CI (0.21 to 0.99), *p* < 0.05). Most of the specific AEs did not vary significantly between responders and non-responders. However, itchy and red eyes (*n* = 8, 9%, for both) were reported only in the responder subgroup (χ^2^_(1)_ = 6.9, *p* < 0.01 for both). Additionally, dry mouth was reported at higher rates among the responders (*n* = 9, 10%) than by non-responders (*n* = 2, 4%) (χ^2^_(1)_ = 3.9, *p* < 0.05).

In descending order of frequency, reported AEs included central nervous system AEs (*n* = 33, 23%), psychological AEs (*n* = 21, 14%), ophthalmic AEs (*n* = 16, 11%), gastrointestinal AEs (*n* = 15, 10%), musculoskeletal AEs (*n* = 11, 8%), cardiovascular AEs (*n* = 10, 7%), and auditory AEs (*n* = 9, 6%).

We further evaluated the associations between MC administration routes and AEs. There were no significant differences between patients reporting MC-related AEs and MC administration routes (i.e., inflorescence, oil extract, or a combination of these administration routes) (0.08 95% CI (0 to 0.25); *p* = 0.59). Additionally, no differences were observed between the different consumption methods (e.g., smoking, vaping, sublingual etc., *p* > 0.05).

### 3.4. MC Treatment Complexity

**Figure 2:**
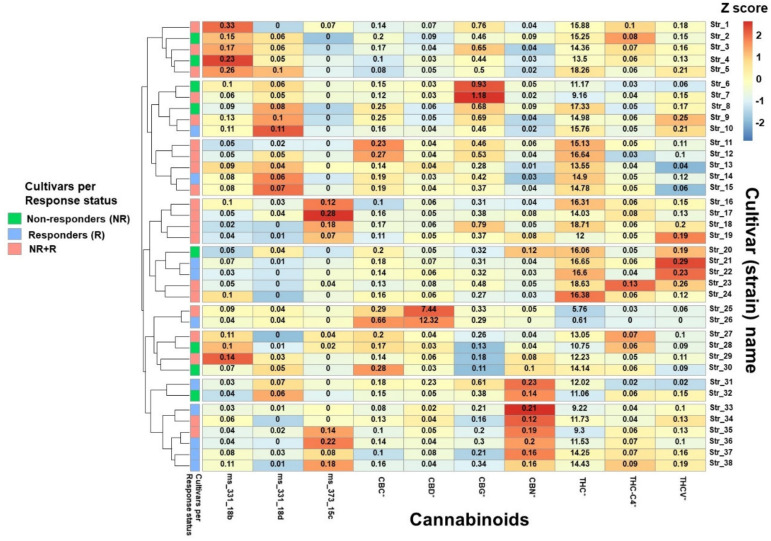
Relative phytocannabinoid concentrations in the most frequently consumed cultivars. Colors on the graph represent the scaled phytocannabinoid concentration variations between cultivars; the numbers in each box represent the concentration (% w/w) of the specific phytocannabinoid within each cultivar. Note: * for each phytocannabinoid, the concentrations of the acid and its neutral counterpart were summed and reported as the total content; Method used: package “pheatmap”, function pheatmap, with the “Euclidean” (default) distance measure used in clustering rows, “complete” clustering method used on *z*-scored data scaled by row. Note: THC, (-)-Δ^9^-*trans*-tetrahydrocannabinol; CBD, cannabidiol; CBC, cannabichromene; CBG, cannabigerol; CBN, cannabinol; THC-C4, (-)-Δ^9^-*trans*-tetrahydrocannabinol-C4; THCV, (-)-Δ^9^-*trans*-tetrahydrocannabivarin.

The complexity of MC treatment in Israel is due to the variety of available cultivars in Israel (about 100 different cultivars or “strains”) and the options for patients to consume more than one cultivar in the same month, with varying doses of each cultivar. Consequently, the 68 patients in the current study reported consumption of 50 different MC cultivars combinations were reported in the current study by the 68 patients we had full cultivar lab information on. Notably, 46 (92%), 1 (2%) and 3 (6%) of the 50 possible combinations were compiled of cultivars that were THC-, CBD-dominant or contained equally high contents of THC:CBD, respectively. These 50 combinations comprised 38 unique cultivars. Figure 2 shows a *z*-score clustered heatmap of the main phytocannabinoids (presented as total concentrations in % w/w) in the 38 cultivars consumed by the sample subgroup. Based on the phytocannabinoid concentration variability, these cultivars were clustered to nine different groups. Figure 2 also shows that in the combinations of cultivars consumed, ten cultivars were consumed only by responders, eight cultivars were consumed only by non-responders, and the rest of the cultivars (*n* = 20) were consumed by both groups.

### 3.5. MC Treatment Characteristics

In this subgroup analysis we included data only from patients who smoked or vaped MC inflorescences and not those who consumed oil extracts sublingually, in order to avoid comparing between different routes of administration (different pharmacokinetics). Since the inflorescences in this study were analyzed in their natural form, monthly consumption of phytocannabinoid doses were calculated according to total phytocannabinoid concentrations rather than analyzing separate acid or neutral concentrations, in order to simulate the neutral maximum content of phytocannabinoids consumed following smoking or vaporization. This calculation corrects for any differences that may arise in phytocannabinoid profiles as a result of decarboxylation due to mishandling or storage of the MC inflorescences. Thus, the minority of patients that reported sublingual consumption of oil extract (*n* = 12) or combined these with inflorescences (*n* = 19) were not included in this subgroup analysis. Consequently, 68 (47%) patients reported exclusive MC inflorescence consumption via inhalation. Of these, 45 (66%) of them were responders and 23 (34%) were non-responders.

**Figure 3:**
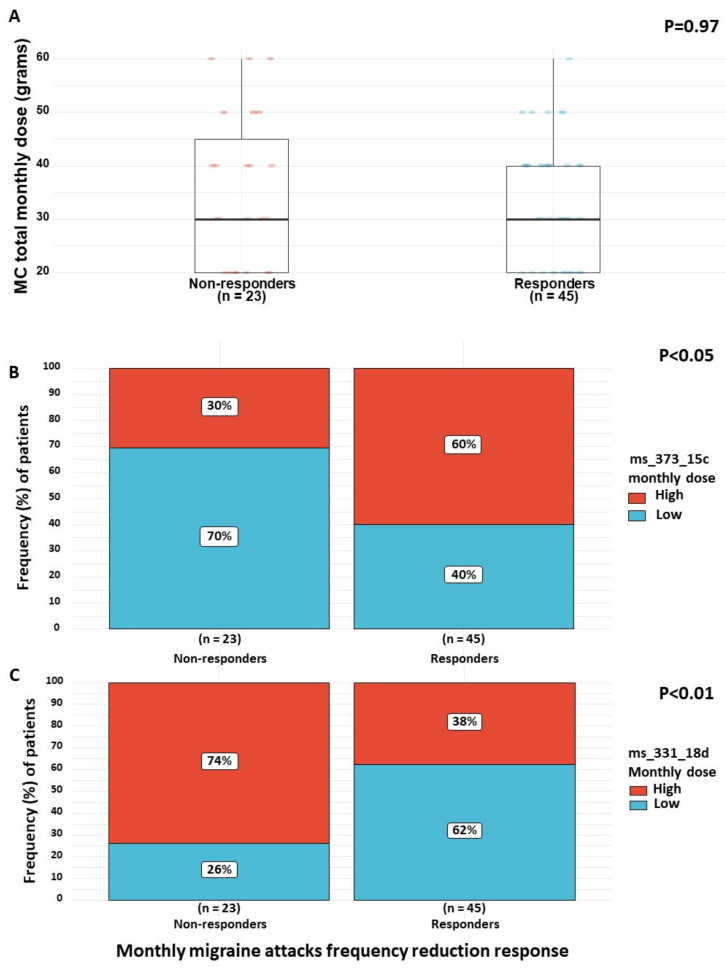
(**A**–**C**) Phytocannabinoid dose differences between responders and non-responders. Note: MC, medical cannabis. Response refers to reduction in the monthly migraine attacks frequency following the initiation of MC treatment (i.e., ≥50%) compared to non-responders (i.e., <50%).

For the abovementioned 68 patients, we first evaluated the differences in total MC monthly dose between responders and non-responders. No significant differences were found (30 (20–40) g and 30 (20–45) g, respectively) (0.25 95% CI (−0.26 to 0.76); *p* = 0.97) (Figure 3A). Therefore, we evaluated the impact of the monthly doses of specific phytocannabinoids. As the distribution of monthly doses of specific phytocannabinoids were non-normal, we separated specific phytocannabinoids into low and high monthly dose groups, based on the distribution of consumption in our patient sample.

We found that responders were more likely to consume high doses (7.9–109.5 mg per month) of the phytocannabinoid ms_373_15c (*n* = 27, 60%) and low doses (0–9.9 mg per month) of the phytocannabinoid ms_331_18d (*n* = 28, 62%) compared to non-responders, who were more likely to consume low doses (0–7.8 mg per month) of ms_373_15c (*n* = 16, 70%) and high doses (10.0–46.8 mg per month) of ms_331_18d (*n* = 17, 74%) (3.40 95% CI (1.10 to 12.00); *p* < 0.05 and 0.22 95% CI (0.05 to 0.72); *p* < 0.01, respectively) (Figure 3B,C). The other phytocannabinoids monthly doses did not vary significantly between the subgroups. Importantly, no differences were found between responders and non-responders in the daily frequency of MC consumption (5 (2.5–7) times per day and 4.5 (3–6) times per day, respectively) (0.18 95% CI (−0.34 to 0.71), *p* = 0.99). Additionally, no differences were found in the number of monthly cannabis cultivars combinations (2 (1–2) cultivars, respectively) (0.04 95% CI (−0.47 to 0.56), *p* = 0.99). Interestingly, among the 38 unique cultivars that patients consumed in their combinations, 12 contained considerable amounts of ms_373_15c and none or very low amounts of ms_331_18d. These cultivars appeared more frequently among the responders (42 appearances in cultivar combinations) than the non-responders (14 appearances in cultivar combinations).

### 3.6. Migraine Treatment Characteristics

A total of 65 (45%) of the patients reported any current consumption of pharmaceutical analgesic medications. Although not significant (0.51 95% CI (0.23 to 1.10), *p* = 0.09), more of the non-responders (*n* = 30, 54%) reported consumption of analgesics compared to the responders (*n* = 35, 39%). Nonetheless, there was a significant difference in the type of analgesic intake between the two groups. Non-responders consumed significantly higher rates of weak opioids (*n* = 13, 23%; e.g., tramadol hydrochloride, buprenorphine hydrochloride, etc.), strong opioids (*n* = 14, 25%; e.g., oxycodone hydrochloride, fentanyl, etc.), and triptans (*n* = 9, 16%; e.g., sumatriptan, rizatriptan, etc.) compared to responders (*n* = 4, 5%; *n* = 7, 8% and *n* = 4, 5%, respectively) (0.15 95% CI (0.03 to 0.53); *p* < 0.005, 0.25 95% CI (0.07 to 0.72); *p* < 0.005 and 0.24 95% CI (0.05 to 0.93), *p* < 0.05). No statistically significant variations were found between responders and non-responders in the consumption rates of over-the-counter analgesics, NSAIDs, anticonvulsants, antidepressants, and antiemetics.

## 4. Discussion

In this cross-sectional study, we evaluated patient reports on the frequency of their monthly migraine attacks, both pre- and post-MC treatment. Patients were classified as responders if they reported greater than 50% reduction in monthly migraine attacks post-MC treatment. As expected, responders reported lower current migraine disability and lower negative impact compared to non-responders.

A recent retrospective study conducted by Rhyne et al. (2016) showed that migraine patients who inhaled MC had a significant reduction in migraine frequency [30], which is in line with the results demonstrated here, and supports our finding of high rates of patient reporting of migraine frequency reduction. Migraine is classified as a pain condition. Mechanistically, endocannabinoids have been shown to have an inhibitory effect on serotonin receptors in vivo [31], which is shown to modulate pain and emetic responses. Additional in vivo data showed that THC induced an antinociception effect on the periaqueductal gray matter [32], which is believed to be involved in migraine pathophysiology [33]. Moreover, relatively low levels of the endocannabinoid anandamide (AEA) in the cerebral spinal fluid (CSF) were found to be associated with the mechanism of migraine initiation [11]. A reduction in pain in in vivo models following endocannabinoid [31] and cannabinoid [32] treatments supports our finding regarding a reduction of migraine disability in the responders group. Nonetheless, these studies still do not incorporate all the complexities of whole-plant cannabis treatment.

In this study, responders reported better migraine disability status, less negative headache impact, and better sleep quality. Whilst this result is logical, conflicting results were reported in another cross-sectional study, which demonstrated an association between improved headache disability and migraine intensity, but found no such association with headache frequency [34]. Taken together, our findings suggest that improved migraine disability status and negative impact among MC treatment responders might be attributed directly to MC treatment effects, rather than being secondary to the reduction of the frequency of migraine attacks. Here, we also reported an association between patients with poor sleep quality and less responsiveness to MC treatment in reducing the frequency of migraine attacks. A previous cross-sectional study demonstrated similar results, showing that even without MC treatment, an association was found between poor sleep quality and higher migraine attack frequency [35]. Thus, it might be suggested that migraineurs that responded to MC treatment and demonstrated a decrease in their monthly migraine frequency also had a concurrent sleep quality improvement. However, due to the current study design, we are unable to conclude whether the reported improved sleep quality can be attributed to the decrease in monthly migraine attack frequency or directly due to MC treatment effects.

There is increasing evidence that MC treatment has opioid-sparing effects [36,37,38,39,40]. Here, we found that responders to MC treatment also reported lower rates of consumption of opioids and triptans compared to non-responders. Both opioids and triptans are usually prescribed for migraine pain relief and not for prevention [6,41]. Thus, although we do not have information regarding the use of these medications prior to MC treatment initiation, this might be an indication that patients that responded clinically to MC treatment substituted this conventional treatment for MC.

In this study, we evaluated the differences in relative monthly dose of phytocannabinoids in each cultivar consumed, in both the responders and non-responders groups. To the best of our knowledge, this is the first study to assess the dose consumption of a wide variety of specific phytocannabinoids administered in combinations of cultivars. By doing so, we were able to elucidate associations between specific cannabinoids consumed over a monthly dose and the clinical response of migraine frequency reduction following MC treatment initiation. The most novel finding of this study was the identification that higher rates of patients that reported significant migraine frequency reduction following MC treatment also consumed higher monthly doses of ms_373_15c and lower monthly doses of ms_331_18d. Our group has previously identified these compounds in both THC- and CBD-dominant chemovars according to LC/MS/MS [18,19], however their absolute structure still needs to be elucidated. According to their MS/MS fragmentation spectra, ms_373_15c and ms_331_18d are acidic and neutral phytocannabinoids, respectively. Additionally, we identified specific cultivars that contain this favorable ratio between those compounds. However, it is important to note that we cannot attribute the anti-migraine effect of MC specifically to these phytocannabinoids, as we are yet to understand whether they are biological active. Nevertheless, we suggest using the presence of these phytocannabinoids to help in choosing specific MC chemovars for further research. Unfortunately, due to the relatively small sample size of patients in this study and a large number of cultivars with variable chemical constituents, translating these findings to the clinical setting will require a larger sample size and a more comprehensive approach. However, the work presented here could be the foundation of such a study to include these “lesser known” phytocannabinoid compounds. Currently, there are no clinical trials on migraine and MC [42]. Previous studies on migraine did not assess the phytocannabinoids mentioned in our study [43], and usually regarded “cannabis” as a single adherent medication [30], therefore disregarding the inherent complexity in MC treatment, with differences in over 90 phytocannabinoids [18] between cannabis cultivars [44].

We also found that the incidence of MC-related AEs was higher among non-responders. This may be explained by responders tolerating MC-related AEs better than non-responders. It could also be explained by the responders’ success during trial-and-error to identify a specific MC chemovar that provided them relief with lower rates of AEs. Nevertheless, due to our study design, we could not corroborate these findings. Future studies should, therefore, investigate the association between MC-related AEs and treatment response a priori. Importantly, none of the patients reported aggravation of migraine AEs as a result of MC treatment.

### Limitations

There are four limitations in the current study. Firstly, our results may have been biased by the small sample size; non-parametric models were used to balance this limitation. Secondly, there may be self-reporting bias. Participants were able to respond to the questionnaire under strict anonymity, ensuring there were no risks that their current treatment plan may be altered by their physician. The questionnaire has also been validated. Thirdly, since we cannot compare the initial indications for which responders and non-responders obtained their MC license, it is possible that the presented results have been biased. Nonetheless, since we identified that chronic neuropathic non-cancer pain was the predominant indication for obtaining MC license, we assumed that it is unlikely that differences between the subgroups exist. Lastly, since the frequency of migraine attacks prior to MC treatment was reported in retrospect, recall bias might have occurred.

## 5. Conclusions

Migraine is currently not indicated for a MC treatment license in Israel. Nevertheless, in some cases it falls under the approved chronic neuropathic non-cancer pain indication, making it possible to study migraine more extensively. In this study, we demonstrated that patients responding to MC treatment also reported less disability and lower conventional anti-migraine medications intake. Additionally, we highlighted the importance of recognizing and analyzing the doses of the pronounced MC constituents consumed by patients, which in turn allowed us to better understand MC treatment associations with reduction in migraine attacks frequency. We also identified specific cultivars that contain the favorable ratio of compounds that were associated with migraine frequency reduction. These results might shed light on the beneficial effects of MC on migraine and motivate future studies to utilize a cannabis cultivar with the specific phytocannabinoids mentioned here. This additional work could validate our results and possibly support making migraine an approved indication for MC license in Israel.

## Figures and Tables

**Table 1:** Demographic and medical cannabis (MC) treatment characteristics.

	Non-Responders N = 56	Responders N = 89	Total N = 145	Statistic (*p*)	Effect Size (CI)
**Measure**	**Number of patients (%)**				
Gender					
Female	35 (62)	62 (70)	97 (67)	0.51 (0.48)	0.73 (0.34–1.6)
Male	21 (38)	27 (30)	48 (33)		
Missing N	0	0	0		
	**Median (IQR)**				
Age (years)	46 (35–54)	44 (34–54)	45 (34–54)	0.08 (0.96)	−0.02 (−0.37–0.32)
Missing N	3	3	6		
BMI	25 (22–27)	25 (22–28)	25 (22–27)	0.13 (0.64)	0.05 (−0.28–0.39)
Missing N	0	1	1		
MC treatment duration (years)	3.5 (2.8–5.2)	3 (2–4)	3 (2.4–4.6)	0.22 (0.09)	0.46 (0.10–0.81)
Missing N	4	5	9		
**Measure**	**Number of patients (%)**				
**MC administration route**					
Inflorescence	40 (71)	72 (81)	112 (77)	2.4 (0.31)	0.13 (0–0.29)
Oil extract	7 (12)	5 (6)	12 (8)		
Combination #	7 (12)	12 (13)	19 (13)		
Missing N	2	0			
Inflorescence administration method *					
Pure MC cigarettes	22 (39)	33 (37)	55 (38)	0.04 (0.84)	1.10 (0.54–2.40)
MC cigarettes mixed with tobacco	17 (30)	30 (34)	47 (32)	0.01 (0.89)	0.89 (0.40–1.90)
Bhang	3 (5)	11 (12)	14 (10)	1.1 (0.29)	0.41 (0.07–1.70)
Electronic vaporizer	14 (25)	15 (17)	29 (20)	1.1 (0.29)	0.59 (0.24–1.50)
Manual vaporizer	5 (9)	20 (22)	25 (17)	3.3 (0.06)	2.9 (0.96–10.00)
Missing N	2	1			
Oil extract administration method *					
Sublingual	13 (23)	13 (15)	26 (18)	1.4 (0.24)	0.55 (0.21–1.40)
Swallowing	2 (4)	1 (1)	3 (2)	0.19 (0.67)	0.30 (0.005–5.90)
Missing N	2	1			

# Combination refers to patients consuming MC inflorescence concomitantly with MC oil extract; * administration methods do not add up to 100% due to concomitant routes. Note: CI, confidence interval; IQR, interquartile range; BMI, body mass index; MC, medical cannabis.

**Table 2:** Migraine features.

	Non-Responders N = 56	Responders N = 89	Statistic (*p*)	Effect Size (CI)
**Measure**	**Median (IQR)**			
Age of migraine diagnosis (years)	20 (14–36)	22 (14–32)	0.07 (0.98)	0.07 (−0.27–0.42)
Missing N	1	4		
Average migraine duration (hours)	20 (5.8–35)	15 (5–48)	0.12 (0.72)	0.15 (−0.19–0.49)
Missing N	1	2		
	**Number of patients (%)**			
Activity induced aggravation of migraine	32 (57)	61 (69)	1.20 (0.28)	1.60 (0.73–3.3)
Missing N	1	0		
Unilateral migraine	40 (71)	59 (66)	0.39 (0.53)	0.74 (0.33–1.60)
Missing N	1	0		
Aura+	16 (29)	31 (35)	0.28 (0.60)	1.30 (0.60–2.9)
Missing N	1	0		
Nausea+	25 (45)	51 (57)	1.50 (0.23)	1.60 (0.78–3.40)
Missing N	1	0		
Phono/photo phobia+	38 (68)	60 (67)	0.00 (0.98)	0.93 (0.42–2.00)
Missing N	1	0		

Note: CI, confidence interval; IQR, interquartile range; +, positive for this manifestation.

**Table 3:** Sleep characteristics.

	Non-Responders N = 56	Responders N = 89	Statistic (*p*)	Effect Size (CI)
**Measure**	**Median (IQR)**			
Sleep quality global score (PSQI, 0–21)	11 (9–14)	9 (6–13)	0.30 (0.04)	0.46 (0.03–0.89)
Missing N	22	27		
Sleep latency (minutes)	32 (20–60)	30 (15–60)	0.09 (0.97)	−0.07 (−0.46–0.33)
Missing N	16	21		
Sleep duration (hours)	6.2 (5–7)	6 (5–7)	0.11 (0.92)	−0.09 (−0.49–0.30)
Missing N	16	20		
Subjective sleep quality *	3 (2–3)	2.5 (1–3)	0.18 (0.39)	0.42 (0.02–0.81)
Missing N	15	19		
Sleep latency *	2 (1.8–3)	2 (1–3)	0.15 (0.65)	0.2 (−0.20–0.59)
Missing N	16	21		
Sleep duration *	1 (0–2)	1 (0–2)	0.1 (0.95)	−0.02 (−0.41–0.37)
Missing N	16	20		
Habitual sleep efficiency *	1 (0–2)	0 (0–2)	0.09 (0.99)	0.08 (−0.32–0.49)
Missing N	18	22		
Sleep disturbances *	2 (2–2)	2 (1–2)	0.19 (0.33)	0.59 (0.19–0.98)
Missing N	15	19		
Use of sleeping medication *	1 (0–3)	0 (0–1.2)	0.19 (0.34)	0.35 (−0.05–0.75)
Missing N	17	21		
Daytime dysfunction *	2 (1–2)	1 (1–2)	0.18 (0.40)	0.34 (−0.06–0.74)
Missing N	17	23		

* Components of the PSQI questionnaire global score. Note: CI, confidence interval; IQR, interquartile range; PSQI, Pittsburgh sleep quality index.
